# Performance of the Cellslide^®^ automated liquid-based cytology system amongst HIV-positive women

**DOI:** 10.4102/ajlm.v5i1.278

**Published:** 2016-02-01

**Authors:** Pamela Michelow, Amanda Sherrin, Louise Rossouw, Samson Mohaleamolla, Denise Evans, Avril Swarts, Ntombiyenkosi Rakhombe, Jennifer S. Smith, Cynthia Firnhaber

**Affiliations:** 1 Department of Anatomical Pathology, University of the Witwatersrand and National Health Laboratory Service, Johannesburg, South Africa; 2 Department of Internal Medicine, University of the Witwatersrand, Johannesburg, South Africa; 3 Department of Medicine, University of the Witwatersrand, Johannesburg, South Africa; 4 Right to Care, Helen Joseph Hospital, Johannesburg, South Africa; 5 Department of Epidemiology, University of North Carolina, Chapel Hill, North Carolina, United States; 6 Lineberger Comprehensive Cancer Center, University of North Carolina, Chapel Hill, North Carolina, United States

## Abstract

**Background:**

Many women undergoing cervical screening as part of a national South African screening programme may be positive for HIV. The performance of liquid-based cytology (LBC) on samples from HIV-positive women needs to be determined.

**Objectives:**

The performance of the Cellslide^®^ automated LBC system was evaluated as a possible alternative to conventional cytology in a national cervical cancer screening programme.

**Methods:**

Split samples from 348 HIV-positive women attending an HIV treatment clinic in Johannesburg, South Africa were examined by conventional cytology and monolayer LBC methods. All samples were stained, examined and reported in the same manner. Cytotechnologists were blinded to the conventional smear diagnosis if the LBC smear was screened and vice versa.

**Results:**

The same percentage of inadequate smears (1.4%) was obtained by conventional cytology and LBC. Atypical squamous cells of undetermined significance were observed in 5.2% of conventional smears and 4.0% of LBC smears. Low-grade squamous intraepithelial lesions were found in 35.6% of conventional smears and 32.7% of LBC smears. Only one conventional smear was categorised as atypical squamous cells – cannot exclude a high-grade lesion, whereas five such cases were identified on LBC. High-grade squamous intraepithelial lesions were seen in 21.6% of conventional smears and 23.3% LBC smears. No invasive carcinoma was identified.

**Conclusion:**

The performance of the Cellslide^®^ LBC system was similar to that of conventional cytology in this population of high-risk HIV-positive women, indicating that it may be introduced successfully as part of a cervical cancer screening programme.

## Introduction

Cervical cancer is a significant cause of morbidity and mortality in South Africa. In 2009, it was the second-most common cancer amongst South African women, with an age-standardised incidence rate of 22.33 per 100 000.^1^ Amongst black South African women, cervical cancer was the most commonly histologically diagnosed cancer in 2009, with an age-standardised incidence rate of 26.19 per 100 000.^1^ Unfortunately, more recent incidence data are not available. From a global perspective, an analysis of 187 countries showed that approximately 200 000 women died from cervical cancer in 2010, of which a significant proportion were women aged between 15 and 49 years in under-resourced nations.^2^

In South Africa, 30.2% of women of reproductive age were HIV-positive in 2010, whereas the rate was 29.5% in 2012.^3,4^ The burden of cervical cancer and its precursor lesions is intensified amongst HIV-positive women. In a study from Johannesburg, HIV-positive women had a higher prevalence of cervix lesions related to human papillomavirus (HPV) compared with HIV-negative women, even after controlling for confounding variables such as age and sexual behaviour.^5^ This finding reflects the high rate of co-infection of HIV and HPV. Such co-infection is often associated with higher prevalence of high-risk HPV infections and increased rates of intraepithelial lesions such as high-grade squamous intraepithelial lesions (HSIL), which are often large, multifocal, and have higher recurrence rates. Invasive cervical cancer is also more common amongst HIV-positive women, occurring approximately 10 years earlier and with more rapid progression and poorer prognosis, than amongst HIV-negative women.^6,7^

The national South African guidelines for cervical cancer screening of HIV-positive women recommend screening upon diagnosis of HIV. If the results are negative, follow-up screening every one to three years is recommended. If the initial results show a low-grade lesion (i.e. atypical squamous cells of undetermined significance [ASCUS] or low-grade squamous intraepithelial lesion [LSIL]), a repeat screening is required one year later.^8^ If a high-grade lesion is found (i.e. HSIL or atypical squamous cells – cannot exclude a high-grade lesion [ASCH]), the patient is referred for further evaluation. Suggested screening modalities include conventional and liquid-based cytology (LBC), HPV testing, visual inspection with acetic acid and visual inspection with Lugol’s iodine. A number of studies to compare conventional cytology, HPV testing and visual inspection with acetic acid for screening of HIV-positive women in South Africa are currently underway.^9,10^ It is estimated that 80% of South Africans use public–sector healthcare facilities, including the National Health Laboratory Service. Approximately 1 million screenings using the conventional cervical cytology method were reported by the National Health Laboratory Service in 2013.^11^

LBC is an alternative method of preparing cervical smears for microscopic examination and is widely used in well-resourced nations. However, although it was developed 15 years ago, it has not been adopted into the South African health sector. Several meta-analyses comparing LBC with conventional cytology have reported conflicting results. Abulafia et al.^12^ concluded that a commonly used LBC test was more sensitive and specific compared with conventional cytology for diagnosing cervical dysplasia, whereas Arbyn et al.^13^ and Davey et al.^14^ determined that LBC neither reduces the number of unsatisfactory smears nor improves detection of HSIL. Karnon et al. found that there is uncertainty regarding the ‘relative effectiveness (and cost-effectiveness) of the two main LBC techniques’.^15^ Advantages of LBC include that screenings for HPV and other sexually transmitted infections, including chlamydial and gonococcal infections, can be performed on the LBC collection fluid, without the need for collecting a separate specimen. HPV testing can be performed on the fluid in the vial, and HPV testing or cytology can be performed on the same sample.^16,17^

A large percentage of South African women undergoing routine cervical screening may be HIV-positive.^3,4^ As there is a paucity of literature examining whether LBC can be successfully used to screen for cervical abnormalities in HIV-positive women, the benefit of introducing LBC as part of a national cervical cancer screening programme in South Africa is unclear. The aim of this study was to determine whether Cellslide^®^ (Audit Diagnostics, Cork, Ireland), an automated LBC processing system for the preparation of thin-layer smears, can be used successfully as a screening modality in a high-risk HIV-positive population.

## Research method and design

### Ethical considerations

The study protocol was reviewed and accepted by both the University of the Witwatersrand (Human Ethics Committee) and the University of North Carolina.

### Study design and setting

The study was a non-randomised, prospective, observational evaluation. The study population comprised 348 HIV-positive women involved in a cervical cancer screening study in South Africa.^5^ All participants were enrolled consecutively. Women aged between 18 and 65 years were recruited from an HIV treatment clinic at a tertiary government hospital in Johannesburg, South Africa, between November 2009 and August 2011. Participants were approached for the study whilst in the HIV clinic awaiting medications or an appointment with a healthcare practitioner. Women were ineligible to participate if they were pregnant, had previously undergone a hysterectomy or treatment for cervical neoplasia or cancer, were severely ill or had signs or symptoms suggestive of a sexually transmitted disease. Women who had completed treatment for a sexually transmitted disease were eligible. Women who were menstruating at the time of study enrolment were asked to return within two weeks to participate. The main reasons that women declined to participate in the study were the fear of losing their place in the queue and time constraints.

A cervical fluid sample was taken with a cervical broom. A split-sample method was then used, whereby a conventional cytology smear (Pap smear) was prepared by spreading the collected material onto a glass slide and spray fixing immediately, followed by placing the tip of the brush in a vial containing Cellslide^®^ preservative solution (Audit Diagnostics, Cork, Ireland). Both specimens were processed in the Cytology Unit of the Department of Anatomical Pathology, University of the Witwatersrand/National Health Laboratory Service in Johannesburg, South Africa. The manufacturer’s instructions were used when preparing the Cellslide^®^ thin-layer slide. Both types of sample were stained, coverslipped, examined under the microscope and reported using the Bethesda system for reporting cervical cytology.^18^ The Bethesda system is a widely used cytology reporting system that not only provides guidelines for specimen adequacy but also offers standardised reproducible criteria for cytologic lesions such as ASCUS, LSIL, ASCH and HSIL. The aim of the Bethesda system is to minimise inter-observer variability and facilitate communication between the clinician and the laboratory.^18^

### Laboratory investigations

The conventional and Cellslide^®^ cervical smears were examined by different cytotechnologists. The cytotechnologists reporting the Cellslide^®^ smears were blinded to the conventional smear diagnosis and vice versa. Thirteen cytotechnologists reported out some of the conventional cytology and some of the Cellslide^®^ thin-layer slides. Cytotechnologists participate in stringent internal quality assurance activities. Some of these include all reportedly negative smears undergoing rapid review, all positive smears being evaluated by two technologists and evaluation of each technologist’s ASCUS : SIL ratio. External quality assurance activities employed by this laboratory include the Australian RCPA quality assurance proficiency programme and laboratory accreditation by external accreditation bodies. If quality assurance activities identify suboptimal performance by a cytotechnologist, re-training and intense monitoring of the work quality are undertaken.

### Statistical analysis

Categories were compared using the chi-square test or Fisher’s exact test for proportions. We determined the accuracy of Cellslide^®^ for identifying the diagnostic category correctly compared to the ‘gold standard’ of conventional cytology (Pap smears). The number of positive and negative samples as tested using Cellslide^®^ was compared to the number of samples with and without each cervical abnormality of interest (e.g., HSIL, ASCH, LSIL, ASCUS) as determined by conventional cytology. We calculated the sensitivity, specificity, positive predictive value and negative predicative value for each diagnostic category, as well as the corresponding 95% confidence intervals (CI; binomial distribution assumed). The kappa coefficient was used to test for agreement between diagnostic categories for the split sample. Kappa values < 0.4 were considered to indicate poor agreement, whereas values of 0.41–0.75 were considered to indicate moderate (fair to good) agreement and values > 0.75 were regarded as indicating excellent agreement.^19^ As both methods used the same categories within the rating scale, a weighted kappa coefficient was not required. All analyses were performed at a 5% significance level using SAS version 9.1 (SAS Institute, Cary, North Carolina, United States).

## Results

Very few inadequate smears were obtained by the respective methods (only five [1.4%] for both conventional cytology and Cellslide^®^) (Table 1). A high percentage of abnormal smears were identified with both methods, and a diagnosis of negative for intraepithelial lesion/malignancy (NILM) was found for only 125 (35.9%) by conventional cytology and 129 (37.1%) by Cellslide^®^. LSIL was the most frequently diagnosed epithelial abnormality (124 [35.6%] by conventional cytology and 114 [32.7%] by Cellslide^®^). Both preparation methods diagnosed a substantial number of HSIL cases (75 [21.6%] by conventional cytology and 81 [23.3%] by Cellslide^®^). The Cellslide^®^ method diagnosed an additional six cases of HSIL (*P* < 0.001). ASCUS was diagnosed in 18 samples (5.2%) using conventional cytology and in 14 samples (4.0%) using Cellslide^®^ (*P* < 0.001), whereas ASCH was diagnosed in only one sample (0.3%) using conventional cytology and in five samples (1.4%) using Cellslide^®^ (*P* < 0.014). No cases of glandular lesions or invasive carcinoma were diagnosed by either method.

**TABLE 1:** Results from conventional cytology and Cellslide^®^ automated liquid-based cytology amongst HIV-positive women (*n* = 348) in Johannesburg, South Africa (November 2009 to August 2011).†

Cellslide^®^	Conventional cytology (Pap smear)						Total for Cellslide^®^ *n* (%)

	NILM	ASCUS	LSIL	ASCH	HSIL	Inadequate	
NILM	112	8	9	0	0	0	129 (37.1%)
ASCUS	0	7	4	0	3	0	14 (4.0%)
LSIL	9	3	87	0	15	0	114 (32.7%)
ASCH	0	0	4	1	0	0	5 (1.4%)
HSIL	4	0	20	0	57	0	81 (23.3%)
Inadequate	0	0	0	0	0	5	5 (1.4%)

**Total for conventional cytology**	**125 (35.9%)**	**18 (5.2%)**	**124 (35.6%)**	**1 (0.3%)**	**75 (21.6%)**	**5 (1.4%)**	**348 (100%)**

ASCH, atypical squamous cells - cannot exclude HSIL; ASCUS, atypical cells of undetermined significance; HSIL, high-grade squamous intraepithelial lesion; LSIL, low-grade squamous intraepithelial lesion; NILM, negative for intraepithelial lesion/malignancy.

† Samples were classified according to the Bethesda system for reporting cervical cytology (see ref. 18).

Twenty cases diagnosed as LSIL by conventional cytology were diagnosed as HSIL by Cellslide^®^, and 15 cases diagnosed as LSIL by Cellslide^®^ were diagnosed as HSIL by conventional cytology. Four cases diagnosed as ASCH by Cellslide^®^ were diagnosed as LSIL by conventional cytology. No cases diagnosed as ASCH by one method (either conventional cytology or Cellslide^®^) were diagnosed as HSIL by the other method. Three cases diagnosed as ASCUS by Cellslide^®^ were diagnosed as HSIL by conventional cytology, but no cases diagnosed as ASCUS by conventional cytology were diagnosed as HSIL by Cellslide^®^.

The agreement between the two diagnostic methods was poor for ASCH (*κ* = 33.0, 95% CI: 1.4–33.0) (Table 2). Agreement was also considered poor for ASCUS, because the kappa value was close to the poor–moderate cut-off and the 95% CI was wide (*κ* = 41.1, 95% CI: 18.6–62.5). Agreement was moderate for LSIL and HSIL, and excellent for NILM.

**TABLE 2:** Level of agreement between conventional cytology and Cellslide^®^ for each diagnostic category amongst samples from HIV-positive women (*n* = 348) in Johannesburg, South Africa (November 2009 to August 2011).

Diagnostic category†	Kappa agreement (95% confidence interval)‡
NILM	81.3 (73.3-87.2)
ASCUS	41.1 (18.6-62.5)¶
LSIL	57.9 (47.6-66.7)
ASCH	33.0 (1.4-33.0)
HSIL	65.2 (54.0-74.3)

ASCH, atypical squamous cells - cannot exclude HSIL; ASCUS, atypical cells of undetermined significance; HSIL, high-grade squamous intraepithelial lesion; LSIL, low-grade squamous intraepithelial lesion; NILM, negative for intraepithelial lesion/malignancy.

† Samples were classified according to the Bethesda system for reporting cervical cytology (see ref. 18).

‡ Poor agreement: κ < 0.4; moderate (fair to good) agreement: 0.41 > κ < 0.75; excellent agreement: κ > 0.75.

¶ Agreement for ASCUS was considered poor rather than moderate, because of the wide confidence interval and its proximity to the category cut-off.

For HSIL, Cellslide^®^ showed sensitivity of 76.0% (95% CI: 64.8–85.1) and specificity of 91.0% (95% CI: 87.0–94.2), with a false-omission rate < 7%, compared with conventional cytology (Table 3). In addition, when compared with conventional cytology, Cellslide^®^ showed sensitivity of 89.6% (95% CI: 82.9–94.4) and specificity of 92.2% (95% CI: 87.8–95.4) for NILM, sensitivity of 70.2% (95% CI: 61.3–78.0) and specificity of 87.7% (95% CI: 82.6–91.7) for LSIL, and sensitivity of 100% (95% CI: 2.5–100) and specificity of 98.8% (95% CI: 97.1–99.7) for ASCH.

**TABLE 3:** Diagnostic accuracy of Cellslide^®^ compared with conventional cytology in samples from HIV-positive women in Johannesburg, South Africa (November 2009 to August 2011).

Diagnostic category	Diagnostic performance (n = 343)†		Cellslide^®^			
	
	Cellslide^®^ smears	Conventional smears	Sensitivity (95% CI)	Specificity (95% CI)	Positive predictive value (95% CI)	Negative predictive value (95% CI)
HSIL	81 (23.5%)	72 (20.9%)	76.0% (64.8-85.1)	91.0% (87.0-94.2)	70.4% (59.2-80.0)	93.1% (89.4-95.9)
ASCH	5 (1.5%)	1 (0.3%)	100% (2.5-100)	98.8% (97.1-99.7)	20.0% (0.51-71.6)	100% (98.9-100)
LSIL	114 (33.2%)	124 (36.2%)	70.2% (61.3-78.0)	87.7% (82.6-91.7)	76.3% (67.4-83.8)	83.8% (78.4-88.4)
ASCUS	14 (4.1%)	18 (5.2%)	38.9% (17.3-64.3)	97.9% (95.6-99.1)	50.0% (23.0-77.0)	96.7% (94.1-98.3)
NILM	129 (37.6%)	125 (36.4%)	89.6% (82.9-94.4)	92.2% (87.8-95.4)	86.8% (79.9-92.1)	93.9% (89.9-96.7)

ASCH, atypical squamous cells - cannot exclude HSIL; ASCUS, atypical cells of undetermined significance; CI, confidence interval; HSIL, high-grade squamous intraepithelial lesion; LSIL, low-grade squamous intraepithelial lesion; NILM, negative for intraepithelial lesion/malignancy.

† Five of the original 348 samples were classified as inadequate. Samples were classified according to the Bethesda system for reporting cervical cytology (see ref. 18).

## Discussion

The results show excellent to moderate agreement between conventional cytology and the automated LBC system Cellslide^®^ for diagnosis of NILM, LSIL and HSIL in this population of HIV-positive women. Poor agreement was observed between the two methods for diagnosis of ASCUS and ASCH.

HPV testing is becoming increasingly popular as a primary cervical screening modality. In South Africa, LBC can be efficacious, should HPV testing be implemented. HPV testing and co-testing with either cytology or reflex cytology, if certain high-risk HPV types are found, can be performed on the same vial, without the need for collecting two samples from the same patient. The LBC method may support faster microscope screening and therefore more samples could be screened daily, although not all studies have demonstrated this.^12,13,14,15^ The LBC method is reported to perform better when using computer-assisted screening devices,^12,13,14,15^ which is an advantage given the large number of Pap smears performed in South African public-sector healthcare facilities.

The cost associated with LBC has to be considered. Results on the cost-effectiveness of LBC are conflicting, depending on whether studies found improvements in the adequacy or detection of abnormality rates. A study by Taylor et al.^20^ found that a commonly used LBC product reduced the number of inadequate smears but did not improve detection of histology-confirmed disease (cervical intraepithelial neoplasia grade 1 or worse) and therefore concluded that the increased cost does not justify the implementation of LBC. In contrast, a review by Cox^21^ showed that LBC was cost-effective. A study by de Bekker-Grob et al.^22^ similarly determined that LBC can be cost-effective as a cervical screening modality, specifically if the cost of LBC exceeded the cost of conventional cytology by less than €3.2, the sensitivity of LBC was at least 3% – 5% greater than conventional cytology and the rate of inadequate smears on conventional cytology was at least 16.2%.

Only one study has investigated the performance of LBC on samples from HIV-positive women.^23^ The findings need to be considered critically to inform a decision about introducing LBC in the South African public health sector, as a large percentage of South African women eligible for cervical screening may be HIV-positive.^6,7,24^ The study by Swierczynski et al.^23^ concluded that conventional cytology and LBC detected the same number of squamous intraepithelial lesions. However, the diagnosis of ASCUS by LBC was more likely to be associated with a squamous intraepithelial lesion on follow-up compared with diagnoses of ASCUS by conventional cytology. They also determined that both methods could readily identify infectious organisms. In the current study, the conventional cytology and Cellslide^®^ methods detected a similar number of unsatisfactory smears and ASCUS and LSIL diagnoses, but more cases of ASCH and HSIL were diagnosed by Cellslide^®^. Furthermore, we found excellent agreement between the two methods for diagnosis of NILM and moderate agreement for diagnosis of LSIL and HSIL. Our finding of poor agreement for diagnosis of ASCUS and ASCH highlights the well-described poor intra- and inter-observer reproducibility for these epithelial abnormalities.^25,26,27^ However, it is important to note that the small number of samples in the ASCUS and ASCH categories could have compromised the accuracy of the statistical analysis.

The results of the current study show that Cellslide^®^ has good sensitivity and specificity for NILM. As a screening test, a negative result is useful for determining that the patient does not have the disorder. At this initial screening, Cellslide^®^ correctly identified more than 90% of samples that showed no cervical abnormalities. Similar to these results, a Brazilian study found that the agreement between conventional cytology and LBC was highest in the NILM category.^28^ The authors further noted that this influenced the agreement rate, as the majority of cervical smears were negative. In comparison, the proportion of samples that showed abnormal cytology exceeded 60% for both preparation methods in the current study. Other studies have also documented below-excellent agreement for epithelial abnormalities when comparing conventional cytology to LBC preparations.^27,28^ Factors that influence agreement include the method employed to collect the smear, variations in cellular material between the conventional and LBC samples and the level of experience of the cytotechnologists in interpreting the smears.^29,30,31,32,33,34^

### Limitations

One of the main limitations of the current study is the small number of samples in some of the epithelial abnormality categories (e.g., ASCUS, ASCH) as determined by the Bethesda system; these results should be interpreted with caution. Another limitation is that cellular morphology is somewhat different on LBC preparations compared with conventional cytology and cytotechnologists face a learning curve when moving from conventional cytology to LBC.^30^ In addition, training in LBC cytomorphology is recommended to facilitate accurate interpretation of an LBC smear.^34^ As this study was conducted over only a limited time period, cytotechnologists may not have mastered LBC cytomorphology completely. Accurate costing of a cervical smear, whether for conventional cytology or LBC, is a complex problem and beyond the scope of the current study. However, cost is a critical factor and must be considered when deciding whether to move to LBC or continue using conventional cytology.

### Conclusion

Results obtained with Cellslide^®^ were similar to those of conventional cytology in this population of high-risk HIV-positive women. The technique may therefore be used successfully should it be decided to move to LBC.
